# Anesthetic Challenges for a Pediatric Leukemia Patient Undergoing Total Body Irradiation (TBI): A Case Report

**DOI:** 10.7759/cureus.53535

**Published:** 2024-02-04

**Authors:** Mohsin N Butt, Nazia Shamim, Asma Faraz

**Affiliations:** 1 Department of Anaesthesiology, Aga Khan University Hospital, Karachi, PAK

**Keywords:** leukemia, pediatric anaesthesia, anaesthesia, total body irradiation, radiotherapy (rt)

## Abstract

Radiotherapy of the whole body is called total body irradiation (TBI). It is a well-established component of conditioning regimens before stem cell transplantation in juvenile leukemia. The patient was a three-year-old child with a diagnosis of B-cell acute lymphoblastic leukemia and planned for stem cell transplantation. He was given TBI under anesthesia for three consecutive days prior to the bone marrow transplantation under general anesthesia. The important concerns were related to neutropenia/immune suppression, parental consent for repeated anesthesia, nothing per oral guidelines for the TBI treatment, the possibility of high-grade fever, high chance of respiratory tract infections with repeated anesthesia exposure, etc. Proper preparation, teamwork, and collaborative efforts and the child’s parents made this treatment possible with intended success.

## Introduction

Radiotherapy of the whole body is called total body irradiation (TBI). It is a well-established component of conditioning regimens before stem cell transplantation in juvenile leukemia [1]. The two objectives of the myeloablative conditioning regimen are to eliminate leukemic cells and to prevent graft rejection by suppressing the recipient's immune system [2]. Prior to the hematopoietic stem cell transplant (HSCT), TBI along with the chemotherapeutic drugs are used to suppress the recipient's immune system and ablate the patient's hematopoietic stem cells [3]. TBI-containing conditioning regimens before a scheduled pediatric stem cell transplantation have been proven to be highly effective [1]. The TBI treatment is usually delivered twice a day for three to four consecutive days. The usual gap of TBI treatment per day is six hours. In pediatric patients, anesthesia or sedation is required to conduct this very crucial treatment effectively.

It was presented as an e-poster in the 4th National Anaesthesia Research Symposium during 8-10 September 2023, at the Aga Khan University Karachi, Pakistan. The Abstract was published in Anesthesia and Analgesia [4].

## Case presentation

A three-year-old child was presented to the radiation oncology department with a diagnosis of B-cell acute lymphoblastic leukemia (high risk) and planned for stem cell transplantation. He was planned to deliver TBI under anesthesia for three consecutive days prior to the bone marrow transplantation. A preoperative anesthesia assessment was done, and the parents were counseled about the pre-anesthetic fasting protocol and potential complications related to repeated anesthesia exposure. After parental consent, all necessary arrangements were made in the radiotherapy suit. For the first treatment session of each day, nothing per oral period was six hours for solid and semi-solid food. In the second treatment session of each day, clear liquid (i.e., water, non-pulpy apple juice, and clear lollipop candy) was allowed up to two hours prior to the second anesthesia induction of the day. Standard monitoring, including electrocardiogram, non-invasive blood pressure, oxygen saturation, and temperature, were applied. Inhalational induction was done through sevoflurane 8% with 100% oxygen. The porta catheter was already in place, so no other intravenous access was made. Once the patient attained the desired depth of anesthesia, a laryngeal mask of size 2 was inserted.

**Figure 1:**
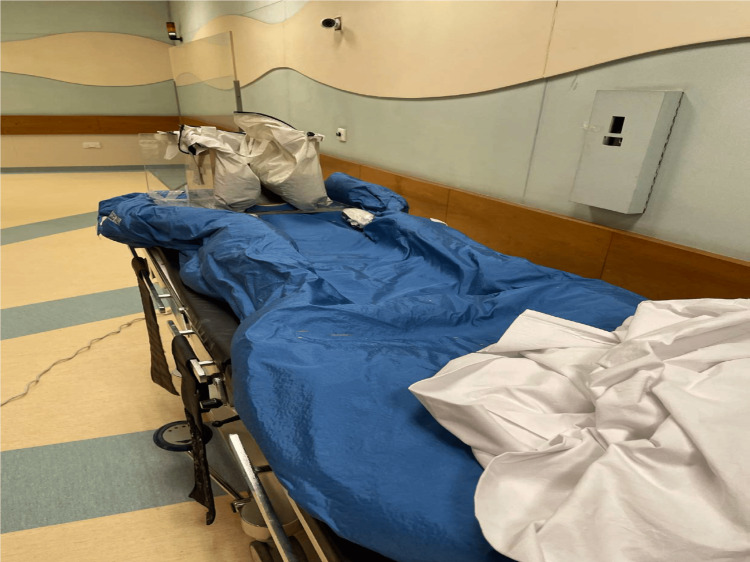
Predesigned couch with a transparent box on the side of the head to place the patient

In the maintenance phase, sevoflurane was set at a level of 2% with 100% oxygen. Ventilation was assessed through the end-tidal carbon dioxide monitoring targeting a level of 40-50 mmHg. The patient was placed in a predesigned case (Figure 1) and the procedure table was kept at a uniform distance of 300 cm from the radiotherapy machine. The head of the patient was covered with a transparent plastic box and the walls of the box were filled with a material with a density similar to human body tissue. Monitors were focused by the already installed closed-circuit camera in the radiotherapy room. The average duration of each treatment period (i.e., beam time) was about 20-25 minutes. During the treatment period, the patient was monitored through the CCTV camera from the control room of the radiotherapy suit. A similar sequence of anesthetic management was repeated six times (twice a day) over a period of three days. Each day, the target gap between the two treatments (beam exposure) was six hours. The whole treatment of TBI (all six cycles) delivered under anesthesia remained uneventful. Each time, the patient was shifted to the post-anesthesia care unit (PACU) adjacent to the radiotherapy suit for an average period of 45 minutes.

## Discussion

The common risks associated with non-operating room anesthesia (NORA) are unfamiliar work environment, insufficient lighting of the NORA places, noise, restricted access to the patient, unfamiliar equipment and team members [5]. The challenging concerns are the positioning of the patient, airway access during the procedure, compatible monitoring, developing intravenous access, delivery of anesthesia/sedation, and management of complications in non-operating room suites [6].

It was a specialized procedure in a diseased child preceding bone marrow transplantation. The anesthesia was planned and delivered in a non-operating room environment. There were multiple anesthesia concerns related to the conduct of TBI in the radiotherapy suite. Along with the general concerns of the pediatric population, there were a few very distinguished anesthetic concerns. The important concerns were related to the neutropenia/immune suppression, parental consent of repeated anesthesia, nothing per oral (NPO) guidelines for the TBI treatment, the possibility of high-grade fever after the first day of treatment, the possibility of pancytopenia (i.e., low hemoglobin and platelet count), a high chance of respiratory tract infections with repeated anesthesia exposure, etc. All the above concerns were monitored and should be taken care of with all possible precautions.

Neutrophils, which are the most prevalent form of white blood cell, play a crucial first line function in innate immune defense. Patients who experience chemotherapy-induced neutropenia are much more vulnerable to invasive bacterial or fungal illness [7]. Numerous anesthetics inhibit different aspects of human neutrophil function, but the underlying mechanisms are still poorly understood [8]. Parental understanding of the necessity of treatment versus disadvantages related to repeated anesthesia exposure was the most important concern for the proper delivery of TBI. It has been reported in the literature that children who underwent repeated surgeries under general anesthesia were at risk for later behavioral and emotional issues [9]. Another important concern was post-radiotherapy febrile neutropenia. In one published study, the frequency of febrile neutropenia after total body irradiation was 37% of patients [10]. Respiratory tract infection is also a relatively common adverse effect with an estimated overall frequency of 24% of patients in a single-center study [10].

As per the standard pediatric nothing per oral (NPO) guidelines before general anesthesia, some safe modification may be required to conduct this very important, time-sensitive, and outcome-oriented treatment (i.e., TBI). Emotional and ethical aspects must also be considered along with pure clinical instructions. The child who is planned for twice-daily anesthesia exposure (six hours apart) must have complete NPO before the first anesthesia exposure of the day. However, after the complete recovery from the first anesthesia, the child needs to be calmed down by giving multiple un-harmful meal options like dextrose containing clear liquids, non-pulpy fruit juices, etc. We arranged clear lollipop candies for the child, and it was successfully satisfying for the child until his second anesthesia of the day. A routine diet was allowed after complete recovery from the second anesthesia. Chances of acquiring respiratory tract infection were also very high in patients undergoing TBI treatment, partially due to the exposure to TBI and an additional risk is also added due to the repeated anesthesia exposure and airway manipulation. Although the anesthesia was administered through supraglottic airway devices, the chances of post-procedure respiratory tract infection remain high in such types of patients. Therefore, the patient needs a thorough history and clinical examination before each anesthesia administration. The risks and benefits of treatment must be weighed to decide to complete the scheduled regimen of TBI. The primary oncologist, radiation oncologist, and anaesthesiologist must be in close communication for optimal decision-making.

## Conclusions

Anesthesia administration in TBI procedures requires an in-depth history of the patient and clinical examination to plan for possible risks that may occur during the treatment. Therefore, proper planning, coordinated preparation, teamwork, and good collaborative efforts of anaesthesiologists, radiation oncologists, hematologists, radiotherapy physicists, allied health staff, and, last but not least, the child’s parents make this treatment possible with intended success.
